# Case Report: Two cases of primary malignant melanoma of the female genital tract and literature review

**DOI:** 10.3389/fimmu.2026.1797752

**Published:** 2026-03-31

**Authors:** Qi Li, Jia Zeng, Xidie Li, Meiyuan Huang, Yuqing Wu, Jinjin Wang, Huan Chen

**Affiliations:** 1 Department of Obstetrics and Gynecology, Zhuzhou Central Hospital, Zhuzhou, Hunan, China; 2 Department of Pathology, Zhuzhou Central Hospital, Zhuzhou, Hunan, China; 3 Department of Obstetrics and Gynecology, Hunan Provincial Maternal and Child Health Hospital, Changsha, Hunan, China

**Keywords:** bevacizumab, case report, immunotherapy, PD-L1, primary cervical malignant melanoma, tislelizumab

## Abstract

Primary malignant melanoma of the cervix (PMMC) is an exceptionally rare and aggressive malignancy with a poor prognosis. Due to its rarity, there are no standardized treatment guidelines. This case report presents two patients with PMMC, contributing to the literature through a novel treatment approach and literature review. Both patients presented with vaginal bleeding: one postmenopausal without obvious cervical pigmentation, the other premenopausal with a brown, friable cervical mass. Histopathology confirmed PMMC in both. The first patient underwent radical surgery without adjuvant therapy and died from recurrence 36 months later. The second patient received radical surgery, adjuvant chemotherapy, and bevacizumab maintenance, followed by a combination of carboplatin, dacarbazine, and Tislelizumab for pulmonary metastases, achieving significant tumor regression after two cycles. A review of nine reported cases treated with PD-1/PD-L1 inhibitors revealed that radical surgery was performed in 90% of patients, yet 80% did not receive chemotherapy or radiotherapy, and most (80%) still experienced disease progression with immunotherapy, underscoring its limited efficacy. PD-L1 expression, assessed in only three cases (all negative), did not reliably predict response. In conclusion, PMMC remains a formidable clinical challenge with high recurrence rates despite surgery. Response to immune checkpoint inhibitors is inconsistent, but multimodal strategies integrating surgery, chemotherapy, anti-angiogenic agents, and immunotherapy may offer clinical benefit and warrant further investigation to improve outcomes in this aggressive disease.

## Introduction

Malignant melanoma (MM) is a malignant tumor originating from melanocytes in the basal layer of the epithelium. While most frequently involving the skin, a small subset of melanomas arises from mucosal surfaces. Among mucosal melanomas of the female genital tract, which account for only 3–7% of all mucosal melanomas, the vulva and vagina are the most common primary sites (1). Primary malignant melanoma of the uterine cervix (PMMC) is exceedingly rare, representing merely 2–13.3% of female genital tract melanomas and approximately 9% of all cervical malignancies (2). This rarity contributes to a significant clinical challenge, characterized by the absence of early symptoms, non-specific clinical signs, and a lack of awareness among clinicians.

The aggressive biology of PMMC is notable, with a propensity for rapid local invasion and metastatic spread. Consequently, a majority of patients are diagnosed at an advanced stage, leading to a historically poor prognosis and high mortality rate (3). Furthermore, the scarcity of cases has precluded large-scale clinical trials, resulting in a critical lack of standardized treatment protocols or evidence-based management guidelines (4). Current therapeutic strategies are largely extrapolated from experience with cutaneous melanomas, squamous cell carcinoma of the cervix, or from anecdotal case reports.

This report presents two patients with PMMC, details their clinical course, management, and outcomes. The first case involved a 73-year-old woman diagnosed with stage IB1 disease who underwent radical surgery without adjuvant therapy and succumbed to recurrence after three years. The second case involved a 45-year-old woman initially staged as IIA, but whose final pathology was upstaged to IIIC1p. She underwent radical surgery followed by adjuvant chemotherapy combined with bevacizumab maintenance therapy. Following thirteen cycles of bevacizumab maintenance, surveillance imaging detected pulmonary metastases. She was subsequently treated with a systemic regimen of dacarbazine, carboplatin, and Tislelizumab. After two treatment cycles, a significant reduction in pulmonary nodules was observed, indicating a favorable therapeutic response. To our knowledge, this represents the first reported use of bevacizumab maintenance therapy following combination chemotherapy in a patient with PMMC. Notably, despite disease progression during maintenance therapy, the patient achieved a significant reduction in pulmonary metastases after two cycles of treatment with dacarbazine, carboplatin and Tislelizumab, providing valuable evidence supporting the potential role of immunetherapy in advanced PMMC. We aim to contribute these experiences to the limited body of literature on this rare malignancy, highlighting the diagnostic challenges, the unpredictability of its clinical course, and the evolving landscape of systemic therapy in advanced and recurrent disease.

## Case description

### Patient 1

A 73-year-old postmenopausal woman presented to a local hospital on April 24, 2021, with a complaint of vaginal bleeding, occurring 24 years after menopause. Her medical history included well-controlled type 2 diabetes of 20 years’ duration and hypertension of 10 years’ duration, managed with daily oral metformin hydrochloride, gliclazide, and amlodipine besylate. There was no history of hormone therapy, prior surgery, radiotherapy, chemotherapy, tobacco use, alcohol consumption, or family history of malignancy.

On April 25, 2021, a ThinPrep cytologic test (TCT) showed atypical squamous cells of undetermined significance (ASC-US). Human papillomavirus (HPV) testing was positive for type 33. Cervical biopsy and endocervical curettage (ECC) were performed on April 28, 2021. The pathology report, available on May 6, 2021, indicated malignant tumor involving the cervix at the 2 o’clock position, the endocervical canal, suggestive of a poorly differentiated carcinoma. Immunohistochemistry (IHC) revealed: CK(-), CK7(-), P16(+++), P40(-), HMB45(focal+), CK5/6(-), Syn(-), CgA(-), S-100(+++), Melan-A(+++), CD3(-), CD20(-), and Ki-67(+70%). These findings were consistent with a diagnosis of malignant melanoma.

The patient was subsequently referred to a provincial hospital on May 8, 2021. Physical examination upon admission did not reveal any abnormal pigmented lesions on the skin or mucous membranes. Gynecologic examination showed no pigmentation in the vagina or on the cervix. The cervix appeared atrophic and firm, with no parametrial induration. A clinical diagnosis of stage IB1 primary cervical malignant melanoma was made.

On May 12, 2021, she underwent open abdominal radical hysterectomy and bilateral salpingo-oophorectomy without pelvic lymphadenectomy. Final pathology confirmed cervical malignant melanoma with invasion into the fibromuscular layer of the cervix (depth >1/3 but <2/3). Lymphovascular space invasion was absent. The tumor did not extend upward into the uterine cavity or downward into the vaginal fornices, and all vaginal surgical margins were free of involvement. IHC of the surgical specimen demonstrated HMB45(+), Melan-A(+), S100(+), Ki-67(+70%), CK-pan(-), and EMA(-).

For economic reasons, the patient elected not to receive any adjuvant therapy postoperatively and was followed with observation alone. She died 36 months after surgery due to tumor recurrence.

Timeline:

**Table:** 

Date	Event
2001~	Diagnosis of diabetes
2011~	Diagnosis of hypertension
2021-4-24	presented to local hospital for vaginal bleeding
2021-4-25	TCT showed ASC-US
2021-4-28	underwent cervical biopsy and ECC
2021-5-6	diagnosis of malignant melanoma
2021-5-8	referred to provincial hospital
2021-5-12	radical hysterectomy and bilateral salpingo-oophorectomy
2024-6	died due to tumor recurrence

### Patient 2

A 45-year-old woman, gravida 3 para 2, was admitted to our hospital on August 1, 2024, with a one-month history of abnormal vaginal bleeding, which had begun on June 29, 2024. Prior to referral, she visited a local hospital on July 19, 2024, where pelvic ultrasound identified a solid, echogenic cervical lesion measuring approximately 36×43×27 mm with irregular contours and ill-defined margins. ThinPrep cytology (TCT) and human papillomavirus (HPV) testing at that time were unremarkable. The patient reported regular menstrual cycles and denied any history of abnormal vaginal discharge or postcoital bleeding. Her past medical, family, and psychosocial histories were non-contributory.

**Figure 1:**
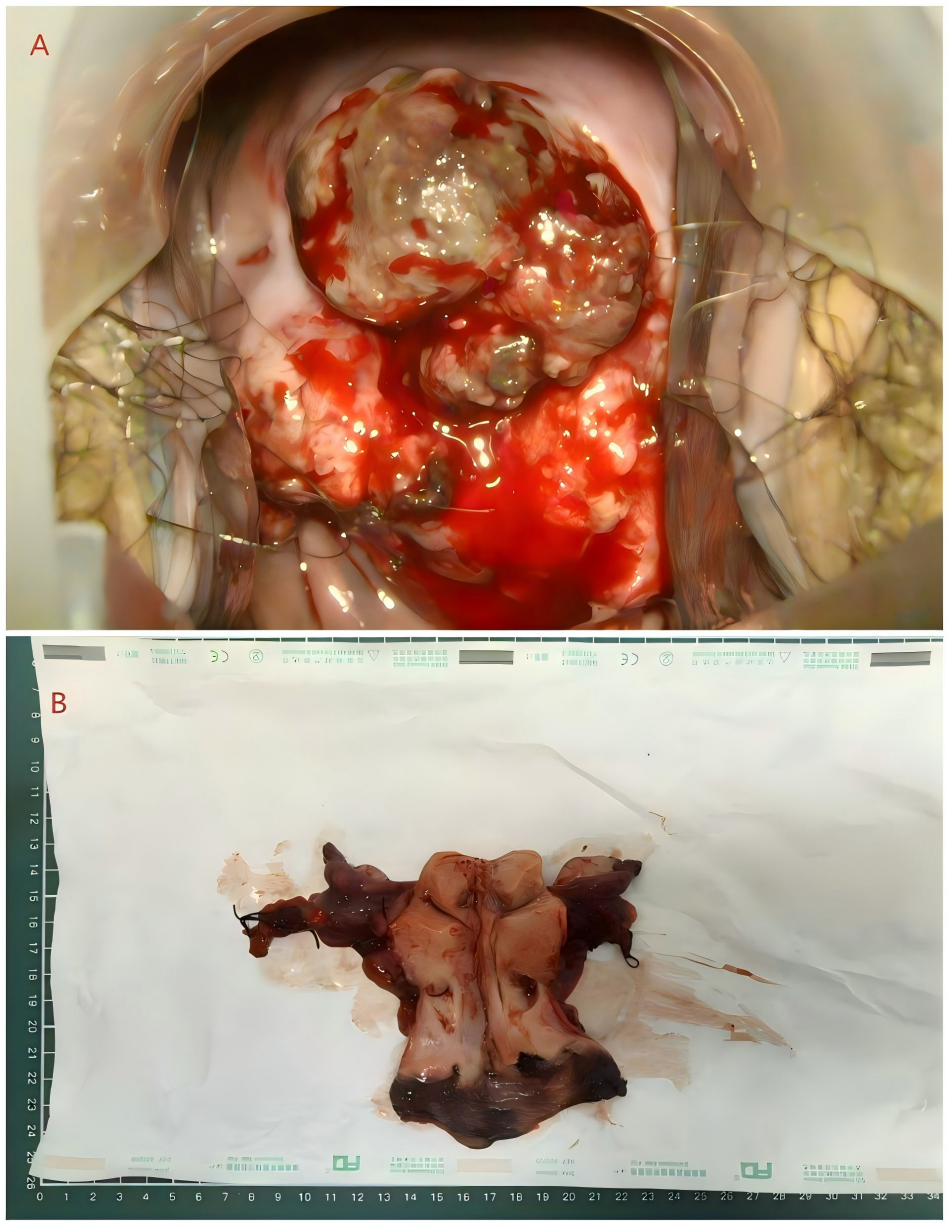
Colposcopy image and Postoperative gross specimen image. **(A)** The colposcopy image before surgery. An egg-sized, brown, friable mass with a rough surface was observed on the cervix. **(B)** Postoperative gross specimen. There was a blue-black mass on the cervix. Multiple blue-black plaques were also observed in the vaginal wall.

Physical examination upon admission was unremarkable. Gynecologic examination revealed a normal vulva. The vagina was patent, with scant bloody discharge and visible pigmentation; no odor was noted. An egg-sized, brown, friable mass with a rough surface was observed on the cervix, which bled on contact (**Figure 1A**). Uterine size and mobility were normal. The left adnexa was palpably thickened, soft, non-tender, and mobile; the right adnexa was normal. No parametrial thickening was detected.

Routine laboratory investigations, including tumor markers (AFP, CEA, CA125, SCC, HE4), were within normal limits. Pelvic magnetic resonance imaging (MRI) demonstrated an occupying lesion (20×9 mm) in the cervix, highly suspicious for cervical carcinoma with possible involvement of the upper third of the vagina. Computed tomography (CT) of the chest, abdomen, and pelvis confirmed a cervical mass (32×30 mm) suggestive of malignancy, with no evidence of distant metastasis.

A biopsy of the cervical mass was performed on August 7, 2024, and histopathological examination confirmed malignant melanoma. Immunohistochemistry (IHC) showed the following profile: CK-pan(–), PAX-8(–), S100(partial+), P40(–), P16/mothers(–), CD45 (LCA)(–), HMB45(+), Melan A(+), Ki-67(+~40%), and vimentin(+). A comprehensive dermatologic, acral, and mucosal survey revealed no additional melanocytic lesions, supporting the diagnosis of primary cervical malignant melanoma (PMMC).

On August 9, 2024, the patient underwent open abdominal radical hysterectomy with bilateral salpingo-oophorectomy and pelvic lymphadenectomy. Intraoperative exploration of the omentum, liver, spleen, and stomach showed no metastatic deposits. Gross inspection of the surgical specimen revealed an enlarged uterus with a smooth serosal surface and a 4 cm blue-black mass on the cervix. Multiple blue-black plaques were also noted along the vaginal wall (**Figure 1B**), leading to an additional circumferential resection of the vaginal cuff. Frozen section of the additionally resected vaginal margin showed no definitive carcinoma.

Final pathology confirmed malignant melanoma involving the cervix and endocervical canal, measuring approximately 2.0×1.6×0.8 cm. Tumor invasion depth was ≥1/3 of the cervical wall with extension into the vaginal fornix. Individual lymphovascular tumor emboli were identified, with focal tumor cells approaching the vaginal resection margin. Metastatic involvement was present in 1 of 12 right pelvic lymph nodes; all four left pelvic nodes were negative (0/4). IHC of the tumor demonstrated: CK-pan(–), S100(+), HMB45(+), Melan A(+), P53(~10% weak+), Ki-67(~30%+), BRAF V600E(+/-), CD117(partial weak+); in depigmented areas, S100 was variably positive and Melan A negative, whereas in pigmented areas, Melan A was positive. The patient was thus classified as FIGO IIIC1p.

**Figure 2:**
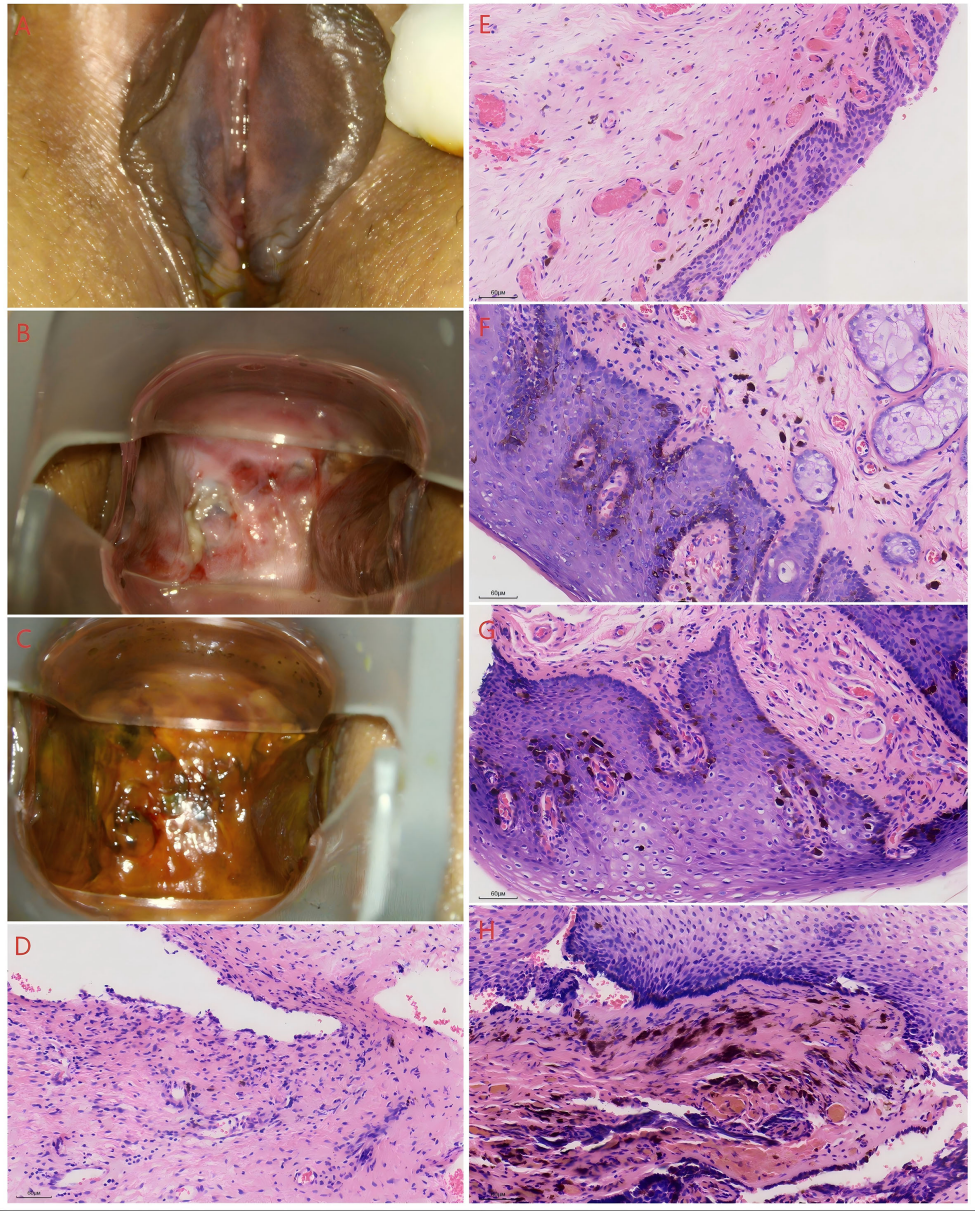
The colposcopy images after surgery and biopsy pathological images. **(A)** labia minora of colposcopy image; **(B)** vaginal stump of colposcopy image(Vinegar test) **(C)** vaginal stump of colposcopy image (iodine test) **(D)** anterior of the vagina stump **(E)** posterior of the vagina stump **(F)** left labia minora **(G)** right labia minora **(H)** right vaginal wall.

The patient received two cycles of adjuvant chemotherapy with carboplatin (AUC 5) plus paclitaxel (175 mg/m²) on August 21 and September 12, 2024, administered at 21-day intervals. On October 31, 2024, colposcopy revealed dark purple pigmentation with acetowhite epithelium on both labia minora, acetowhite epithelium with Lugol’s iodine non-uptake at the vaginal stump edges, and a Lugol’s non-uptake area on the right vaginal wall (**Figures 2A–C**). Biopsies from these sites showed stromal and mucosal melanocytes, raising suspicion for residual melanoma (**Figures 2D–H**); however, tissue samples were insufficient for definitive IHC. At the same day, bevacizumab (5 mg/kg every 14 days) was added to her third chemotherapy cycle. The interval for carboplatin-paclitaxel was extended to 28 days, and she completed a total of four cycles. Serum lactate dehydrogenase (LDH) levels remained between 146 U/L and 173 U/L throughout this period. A follow-up CT scan performed on January 18, 2025 (21 days after the last chemotherapy) showed no evidence of recurrence. She subsequently began maintenance therapy with bevacizumab (15 mg/kg every 21 days). No adverse events occurred.

**Figure 3:**
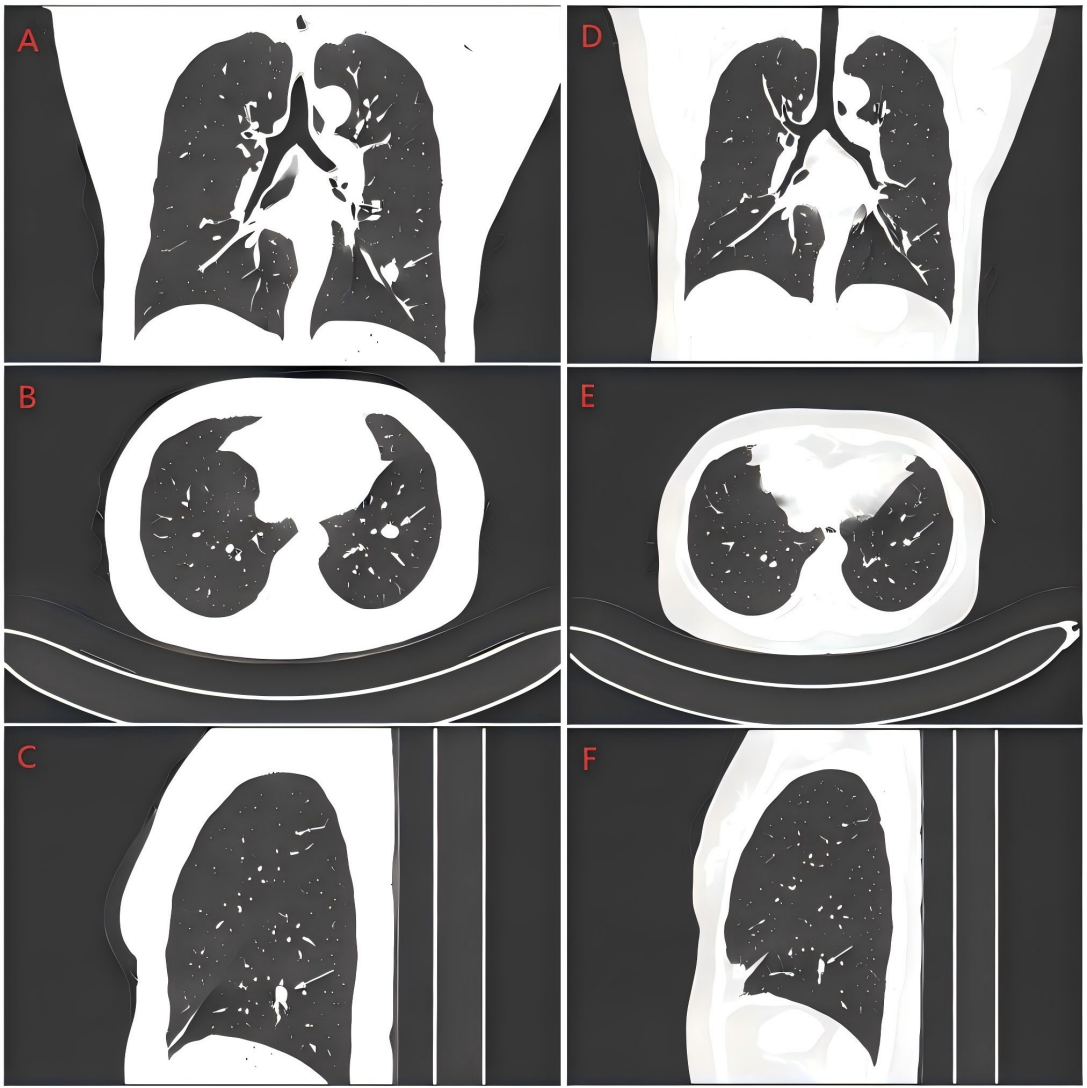
Lung CT images during recurrence and after treatment. **(A)** Lung CT images during recurrence (coronal plane); **(B)** Lung CT images during recurrence(transverse plane); **(C)** Lung CT images during recurrence(sagittal plane). **(D)** Lung CT images after treatment(coronal plane). **(E)** Lung CT images after treatment(transverse plane); **(F)** Lung CT images after treatment (sagittal plane). The white arrow indicates the lesion.

A whole-body CT scan on July 4, 2025, revealed no abnormalities. However, after completing thirteen cycles of bevacizumab maintenance, a surveillance chest CT on November 7, 2025, demonstrated multiple new pulmonary nodules in both lungs. The largest nodule, located in the outer basal segment of the left lower lobe, measured approximately 13.1×9.9 mm, consistent with pulmonary metastases (**Figures 3A–C**). Consequently, on November 9, 2025, systemic therapy was initiated with carboplatin (AUC 5), dacarbazine (1000 mg every 21 days), and Tislelizumab (200 mg per cycle). Following two cycles of this regimen, repeat imaging showed significant shrinkage of the pulmonary nodules (**Figures 3D–F**). The patient has now completed the fourth cycle of treatment with dacarbazine, carboplatin and tislelizumab.

Timeline:

**Table:** 

Date	Event
June 29, 2024	vaginal bleeding
July 19, 2024	ultrasound revealed a mass approximately 36×43×27 mm within the cervix, TCT&HPV(-)
August 1, 2024	Referred to our hospital, Gynecological examination revealed an egg-sized, brown, friable mass with a rough surface was observed at the cervix, with contact bleeding
August 7,2024	the biopsy of the cervical mass confirmed malignant melanoma
August 9, 2024	open abdominal radical hysterectomy and pelvic lymphadenectomy+additional circumferential portion of the vaginal wall
Aug 21, 2024	carboplatin(AUC5) plus paclitaxel(175 mg/m2) chemotherapy
Sep 12, 2024	carboplatin(AUC5) plus paclitaxel(175 mg/m2) chemotherapy
Oct 31, 2024	Colposcopy and biopsy of labia minora, vaginal stump, right vaginal wall.
Oct 31-Dec 28,2024	bevacizumab(5 mg/kg, every 14 days) +carboplatin(AUC5) plus paclitaxel(175 mg/m2)(4 cycles)
Jan 18, 2025	bevacizumab(15 mg/kg, every 21 days)
July,2025	whole-body CT scan detected no abnormalities
Nov 7,2025	whole-body CT scan detected lung metastases
Nov 13,2025	carboplatin(AUC = 5) plus dacarbazine(1000mg/21d) chemotherapy and immunotherapy with Tislelizumab

## Discussion

Primary malignant melanoma of the cervix (PMMC) is an exceedingly rare malignancy that predominantly affects perimenopausal and postmenopausal women with a mean age at diagnosis of approximately 56.5 years, and approximately 85% of patients are postmenopausal (3). PMMC lacks specific clinical manifestations, often leading to delayed diagnosis. Early-stage disease may be asymptomatic, while advanced cases typically present with postmenopausal bleeding, abnormal vaginal discharge, or contact bleeding. In our study, Patient 1 was a 73-year-old postmenopausal woman presenting with vaginal bleeding, without obvious cervical pigmentation, a presentation that can easily be missed. Patient 2, a 45-year-old premenopausal woman, exhibited a typical brownish, friable cervical mass with contact bleeding. Both cases underscore the importance of histopathological confirmation in diagnosing PMMC.

The diagnosis of PMMC relies on histopathological examination supported by immunohistochemistry (IHC). Diagnostic criteria include the presence of melanin in normal cervical epithelium, absence of melanoma elsewhere, junctional activity in the cervix, lentiginous intraepithelial component, absence of epithelial markers (e.g., CK) on IHC, and a pattern of metastasis consistent with cervical carcinoma (5). The most valuable IHC markers are S-100 (sensitive) and HMB-45 (specific); their combination improves diagnostic accuracy, especially in amelanotic variants (6, 7). In our patients, IHC was positive for S-100, HMB-45, and Melan-A, confirming the diagnosis. Notably, Patient 2 presented with multifocal pigmented lesions in the vaginal wall, highlighting the tendency of PMMC to involve the vagina multifocally. This underscores the need for thorough vaginal inspection and, if necessary, wider excision during surgery to achieve clear margins.

There is no standardized treatment protocol for PMMC. Radical hysterectomy with or without lymphadenectomy and/or vaginectomy remains the mainstay of surgical management (8). Radiotherapy is often used for palliation or as an adjunct in cases with positive margins or lymph node involvement. Chemotherapy (e.g., dacarbazine, platinum-based regimens) has shown limited efficacy, with response rates around 15–20% (9, 10).

In recent years, immune checkpoint inhibitors (ICIs), particularly PD-1 blockers such as pembrolizumab and nivolumab, have revolutionized the treatment of advanced melanoma. However, their efficacy in PMMC remains inconsistent and generally poor. We reviewed nine reported cases of PMMC treated with PD−1/PD−L1 inhibitors (9–17) (**Table 1**), which revealed several consistent patterns. Ninety percent of the patients underwent radical surgery, with only one patient not receiving surgery due to being in stage IV. Eighty percent of the patients did not receive chemotherapy, and eighty percent did not undergo radiotherapy. The majority of patients (8/10, ~80%) experienced disease progression despite immunotherapy, with survival ranging from 3 to 13 months, underscoring the generally poor efficacy of ICIs in this population. Only two patients, one reported by Anko et al (12), the other in our study, demonstrated significant tumor regression, suggesting that a small subset may derive clinical benefit. Only three patients underwent PD-L1 immunohistochemistry testing, all of which showed negative expression. PD−L1 expression did not reliably predict therapeutic outcome: two patients exhibited no response, while one patient in the Anko et al. achieved a partial response, implying that additional factors, such as tumor mutational burden (TMB), mismatch−repair status, or features of the tumor microenvironment, likely modulate treatment sensitivity. Our Patient 2 exemplifies the potential of multimodal strategies; after initial surgery and chemotherapy combined with bevacizumab maintenance therapy, she developed pulmonary metastases and was subsequently treated with carboplatin, dacarbazine and Tislelizumab, which led to marked shrinkage of pulmonary nodules. This experience, along with emerging evidence from cutaneous and mucosal melanoma trials, supports further investigation of combination approaches that integrate ICIs with adjuvant therapy to improve outcomes in advanced PMMC (18, 19).

**Table 1:** Clinical information of PMMC patients receiving PD-1 therapy.

Authors	age	Symptom	Figo	Surgery	LM	Chemotherapy	Chemo_regime	Radiotherapy	IHC(PD)	I_time	Survival	Status	Recurrence	Recurrence time	Recur location
2017 (9)	66	VB	IIIA	RH+BSO+PLND+TV	no	yes	Dacarbazine	no	NA	recur	13	dead	yes	6	lung
2018 (11)	40	VD	IIA	RH+BSO+PLND+PV	yes	no	no	no	NA	post	9.5	dead	metastasis		
2019 (12)	54	Asymptomatic	IB1	RH+BSO+PLND	no	no	no	no	Ne	post	50	live	yes	17	Douglas pouch
2019 (13)	61	NA	IIC	RH		no	no	no	NA	recur	9	dead	yes	7	liver,lung
2020 (14)	34	Asymptomatic	IB1	RH+BSO+PLND	no	no	no	no	NA	post	13	dead	yes	9	hepatic and lung
2021 (15)	74	VB	IB1	RH+BSO	no	no	no	no	Ne	post	6	dead	yes	3	lung, liver and pancreatic
2022 (10)	73	VB	IIA1	RH+BSO+PLND	yes	no	no	yes	Ne	recur	7	dead	yes	4	brain and multiple lymph node
2023 (16)	39	VB and VD	IIB	RH+BSO+PLND	no	no	no	no	NA	post	8	dead	yes	4	pelvic
2024 (17)	69	VB and abdominal pain	IVB	No		no	no	yes	NA	direct	3	dead	metastasis		
This Study	45	VB	IIIC	RH+PLND+BSO+PV	yes	yes	carboplatin-paclitaxel+bevacizumab	no	NA	recur	17	live	yes	14	lung

NA, not available; RH, radical surgery; BSO, bilateral salpingo-oophorectomy; PLND, pelvic lymphadenectomy; TV, total vaginectomy; PV, partail vaginectomy; LM, lymphatic metastasis; Ne, negative; I, immunotherapy; recur, after Recurrence; post, post surgery; direct, used directly without surgery.

A recent systematic review by Cuccia et al. highlighted that radiotherapy, in addition to surgery and immunotherapy, may represent a valuable non-invasive treatment option for female genital tract melanomas, particularly in patients who are unfit for surgery or present with locally advanced disease. Although melanoma has traditionally been considered radio-resistant, emerging evidence suggests that modern techniques, such as stereotactic body radiotherapy (SBRT) and hadrontherapy, can deliver high doses per fraction, potentially overcoming this resistance while sparing adjacent healthy tissues. Notably, radiotherapy may also enhance tumor immunogenicity by promoting antigen release and T-cell activation, thereby exerting a synergistic effect when combined with immune checkpoint inhibitors. The review also emphasized the feasibility and efficacy of definitive radiotherapy in vaginal melanoma, demonstrating favorable local control and acceptable toxicity, particularly when combined with immunotherapy (20). Although data on primary cervical melanoma remain limited, the biological rationale and emerging clinical evidence support further investigation into radiotherapy-containing multimodal strategies for PMMC.

This case report provides a detailed clinical account of two PMMC patients, notably illustrating the use of bevacizumab maintenance therapy after surgery and adjuvant chemotherapy, followed by combined chemotherapy and immunotherapy (tislelizumab) upon disease recurrence in the second patient. To the best of our knowledge, this is the first reported case of PMMC in which bevacizumab maintenance therapy was administered following surgery and adjuvant chemotherapy, and was then followed by combined chemotherapy and immunotherapy upon recurrence. The incorporation of a literature review helps situate our observations within the sparse existing evidence. However, the generalizability of our findings is constrained by the inherent limitations of case reports, including the small sample size, single-center origin, and relatively short follow-up for Patient 2. Given the rarity of PMMC, accumulating evidence through continued case reporting and collaborative case series is essential to better understand its clinical behavior and to inform future therapeutic approaches.

## Conclusion

PMMC remains a diagnostic and therapeutic challenge due to its rarity, aggressive behavior, and lack of standardized treatment. Surgery is the cornerstone of management, but recurrence rates are high. PD-1/PD-L1 inhibitors show promise only in a subset of patients, with PD-L1 expression being an imperfect predictor of response. Multimodal approaches, biomarker integration, and international collaboration are critical to improving outcomes in this devastating disease.

## Patient perspective

For Patient 1, the patient and her family were fully informed of the diagnosis and poor prognosis associated with cervical malignant melanoma. They understood the rationale for radical surgery as the primary treatment modality. Postoperatively, the patient elected to forgo adjuvant therapy due to personal preference and concerns regarding potential side effects at her advanced age. Her perspective prioritized maintaining quality of life in the short term. The family expressed appreciation for the clear communication regarding her condition and the surgical care she provided.

For Patient 2, the patient actively participated in the decision-making process throughout her diagnosis and treatment. After the surgery, she was informed of the upstaging to IIICp and the finding of potential residual lesions. She understood the aggressive nature of her disease and provided consent for the combined chemotherapy and bevacizumab regimen. During follow-up, she reported satisfaction with the chosen treatment strategy, noting that she tolerated bevacizumab maintenance therapy well without significant adverse effects such as hypertension or proteinuria. She expressed hope that her clinical course, including the novel use of bevacizumab, would contribute to medical knowledge and potentially benefit future patients with this rare malignancy.

## Data Availability

The original contributions presented in the study are included in the article/supplementary material. Further inquiries can be directed to the corresponding author.
